# Cognitive Trajectories and Subsequent Accelerometer-Measured Movement Behavior in Older Adults

**DOI:** 10.1001/jamanetworkopen.2026.13399

**Published:** 2026-05-19

**Authors:** Mikaela Bloomberg, Laura Brocklebank, Clémence Cavaillès, Aiden Doherty, Séverine Sabia, Andrew Steptoe

**Affiliations:** 1 Department of Epidemiology and Public Health, University College London, United Kingdom; 2 Nuffield Department of Population Health, University of Oxford, United Kingdom; 3 Université Paris Cité, Inserm U1153, Center for Research in Epidemiology and Statistics, Epidemiology of Ageing and Neurodegenerative diseases, France; 4 Faculty of Brain Sciences, University College London, United Kingdom; 5 Department of Behavioural Science and Health, University College London, United Kingdom

## Abstract

**Importance:**

Previous evidence suggests a bidirectional association between physical activity and cognitive function. It remains unclear whether long-term cognitive trajectories are associated with later differences in how older adults allocate time during the day between physical activity, sedentary behavior, and sleep.

**Objective:**

To assess whether long-term cognitive trajectories are associated with the subsequent amount of daily time in physical activity, sedentary behavior, and sleep.

**Design, Setting, and Participants:**

This cohort study included community-dwelling adults aged 50 years or older at baseline and residing in England, using cognitive data from waves 1 (2002 to 2003) to 9 (2018 to 2019) and accelerometer data from wave 10 (2021 to 2023) of the English Longitudinal Study of Ageing.

**Exposures:**

Participant-specific annual rate of change in episodic memory (assessed via immediate and delayed recall) and verbal fluency (assessed via animal naming), estimated using linear mixed-effects models.

**Main Outcomes and Measures:**

The movement behavior composition, comprising mean daily minutes of moderate to vigorous physical activity, light physical activity, sedentary behavior, and sleep time. Differences were estimated in time spent in each behavior between a less favorable memory or fluency trajectory (corresponding to the 25th percentile for participant-specific memory or fluency change), median trajectory (50th percentile), and a more favorable trajectory (75th percentile).

**Results:**

The study included 2529 participants (1394 [55.1%] female, 1135 [44.9%] male) with a mean (SD) baseline age of 56.1 (5.4) years. Compared with the less favorable memory trajectory, the more favorable trajectory was associated with 14 (95% CI, 8 to 21) more minutes of light physical activity and 12 (95% CI, –20 to –5) fewer minutes of sedentary behavior per day, with larger differences among participants older than 70 years (eg, 20 [95% CI, 11 to 30] more minutes light physical activity). Memory-related differences in moderate to vigorous physical activity and sleep were comparatively minor. Fluency showed similar patterns to memory, but time-use differences between trajectories were smaller.

**Conclusions and Relevance:**

In this cohort study of older adults, participants with less favorable long-term memory trajectories were subsequently less active and more sedentary, suggesting that later-life activity patterns may partly reflect cognitive change.

## Key Points

**Question:**

Are long-term cognitive trajectories associated with how older adults later allocate daily time between physical activity, sedentary behavior, and sleep?

**Findings:**

In this UK-based cohort study of 2529 adults aged 50 years or older, those with less favorable memory trajectories over 17 years later spent significantly less time being physically active, with more sedentary time, equivalent to 1.6 fewer hours of light activity per week overall, or 2.3 fewer hours for individuals aged older than 70 years. Differences in sleep time were minor.

**Meaning:**

These findings suggest that in later life, engaging in less physical activity and more sedentary time may partly reflect cognitive decline.

## Teaser

This cohort study assesses the association of cognitive change with later-life activity patterns.

## Introduction

Dementia is a major global public health challenge and one of the leading causes of disability and dependency among older adults,^1^ prompting efforts to identify modifiable risk factors for cognitive decline that might delay its onset. Among the proposed factors are movement behaviors, including physical activity and sedentary behavior (SB). These behaviors have been linked with a range of cognitive outcomes: more physical activity and less sedentary time have been associated with better cognitive performance, slower cognitive decline, and lower dementia risk.^2,3,4,5^

While most research treats movement behaviors as determinants of cognitive health, some evidence also suggests cognitive decline may in turn affect activity patterns.^6,7,8^ Understanding whether cognitive decline precedes physical inactivity is essential for interpreting observational associations between physical activity and cognitive decline to design effective interventions. Identifying predictors of physical activity patterns in later life is important in its own right because declines in these behaviors may signal functional decline, frailty, or loss of independence.^9,10^ However, the relationship between long-term cognitive aging trajectories and subsequent movement behavior remains poorly characterized. One previous study relied on self-report,^6^ which is prone to inaccuracy,^11^ while studies with objectively assessed movement behaviors were small in scale (n = 611) and in highly selected samples^7^ or had short (eg, 4-year^8^) follow-up periods, preventing assessment of long-term cognitive trajectories. These studies also examined each movement behavior separately without accounting for their interdependence across the 24-hour day: along with sleep, physical activity and SB together comprise the 24-hour activity cycle, where more time spent in 1 behavior necessarily comes at the expense of the others.

In the present cohort study, we address these limitations using data from participants aged 50 to 79 years at baseline in the nationally representative English Longitudinal Study of Ageing (ELSA). We modeled individual-specific cognitive trajectories over 17 years, then used compositional data analysis (CoDA) to examine associations between long-term cognitive trajectories and subsequent objectively assessed 24-hour movement behavior patterns comprising moderate to vigorous physical activity (MVPA), light physical activity (LPA), SB, and sleep time.

## Methods

### Data Sources

ELSA is a nationally representative cohort study of community-dwelling adults aged 50 years or older and younger partners living in England. The first wave of data collection occurred in 2002 to 2003. Data were collected biennially until wave 9 (2018 to 2019); wave 10 took place in 2021 to 2023 and wave 11, 2023 to 2024. Details of survey design and administration are available elsewhere.^12^ ELSA received ethics approval most recently from the South Central-Berkshire Research Ethics Committee, with no further ethical approval needed for secondary analysis of ELSA data. Written informed consent was obtained at each interview.

Each wave of ELSA includes a cognitive battery. Wave 10 of ELSA also included an accelerometer substudy, where wave 10 participants were invited to wear an accelerometer for an 8-day period. ELSA respondents aged 50 years or older who participated in at least 1 wave of cognitive testing during waves 1 to 9 with valid accelerometer data at wave 10 were eligible for inclusion in the present analysis. Sample selection is shown in the Figure. For this study, the first wave of cognitive assessment for each participant was considered the analytic baseline. This study followed the Strengthening the Reporting of Observational Studies in Epidemiology (STROBE) reporting guideline.

**Figure.**
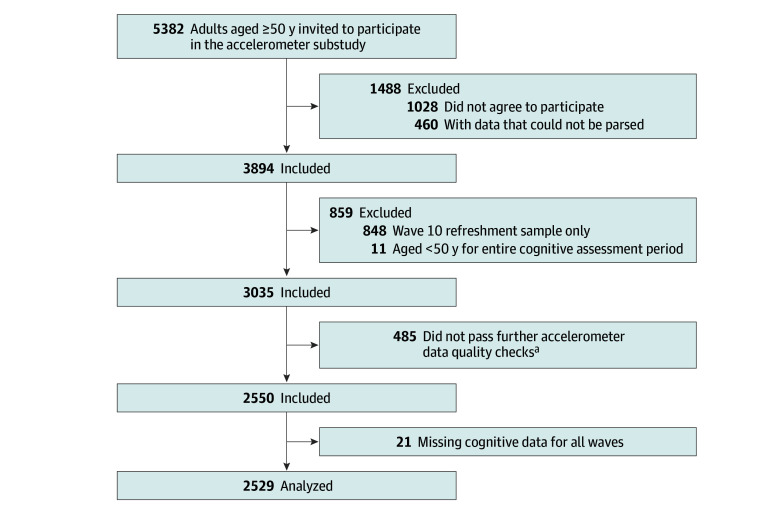
Flowchart of Sample Selection ^a^Participants had to have at least 3 days of data and data within each 1-hour period of the 24-hour cycle. Participants were also excluded if the device could not be calibrated, more than 1% of readings were clipped (ie, fell outside ≥8 gravitational units) before or after calibration, or the mean acceleration was implausibly high (>100 milligravitational units).

### Measurement of Movement Behaviors

During wave 10 data collection, participants were asked to wear an accelerometer on their dominant wrist 24 hours per day for 8 consecutive days. The mean daily time in MVPA, LPA, SB, and sleep time during the wear period was derived from raw acceleration data using algorithmic methods previously applied to large-scale studies including the UK Biobank.^13^ Details are available in eMethods 1 in Supplement 1. Because accelerometer-derived sleep time in this context likely more closely reflects time in bed than sleep duration, we focused our interpretation on waking behaviors (MVPA, LPA, and SB), while presenting sleep results for completeness.

### Cognitive Assessment

Cognitive trajectories were produced using cognitive testing from waves 1 through 9. We excluded cognitive assessments from wave 10 to avoid incorporating scores that in some participants occurred after the accelerometer wear period. The cognitive domains included were episodic memory and verbal fluency, which are important for day-to-day function and show decline with aging and dementia.^14^ Tests of other cognitive domains in ELSA (eg, executive function) were not available at a sufficient number of waves to examine long-term cognitive changes. Memory was assessed using immediate and delayed recall tasks^15^; fluency, the animal naming task.^16^ Cognitive scores were standardized to the analytic sample. Further details of cognitive testing are provided in eMethods 2 in Supplement 1.

### Covariates

Covariates were selected based on their associations with cognitive decline and movement behaviors. They were self-reported and included age at wave 10, and the following baseline measures: sex (male or female), educational attainment (equivalent to less than high school, high school, or above high school), household non-pension wealth standardized by year, smoking status (current smoker or not), alcohol consumption (consumes alcohol or not), physical activity level (engages in MVPA less than weekly, weekly, or more than weekly), self-rated hearing (excellent, very good, good, fair, or poor), depressive symptoms (eMethods 3 in Supplement 1), and diagnosis of the following long-term conditions (yes or no): high blood pressure, diabetes, cancer, heart disease, stroke, and psychiatric conditions.

### Statistical Analysis

We used a 2-stage modeling approach to determine whether cognitive trajectories were associated with subsequent movement behavior patterns. Similar approaches have been applied previously.^17,18^ Detailed statistical methods are available in eMethods 4 in Supplement 1.

In stage 1, we produced individual-specific linear slopes as a summary measure of cognitive decline, derived from linear mixed models fitted separately for memory and fluency. This value reflects the participant’s estimated rate of cognitive change per year. More negative values indicate more rapid cognitive decline. Linear mixed models robustly handled missing data and differences in follow-up duration, assuming data were missing at random.^19^

In stage 2, we applied CoDA to examine associations between memory or fluency decline and the 24-hour composition of movement behaviors (MVPA, LPA, SB, and sleep time) measured at wave 10. CoDA is a well-established method for analyzing compositional data, where the parts sum to a whole (eg, 24 hours in a day),^20,21,22,23^ which involves converting time use behaviors to isometric log-ratios (ILRs) that express the balance of 1 behavior relative to the others. This avoids statistical issues that arise when analyzing raw proportions directly due to their interdependence. Each ILR coordinate was then used as the dependent variable in a separate linear regression model, with individual-specific memory or fluency change as the exposure.

All second-stage models were adjusted for age at wave 10, sex, and the following baseline covariates: educational attainment, wealth, smoking status, alcohol consumption, self-reported physical activity level, self-rated hearing, depressive symptoms, and long-term conditions. Covariates were selected to adjust for potential confounding; plausible mediators between cognitive decline and movement behaviors were not included to avoid overadjustment. Because missingness was minor (<5%) for all covariates and therefore unlikely to substantively bias estimates, missing covariates were singly imputed using predictive mean matching,^24^ with imputation models including all covariates and primary exposure and outcome variables. To facilitate our focus on cognitive decline, we also adjusted for memory or fluency performance at baseline, after determining that in fully adjusted models there was no evidence of interaction between performance at baseline and individual-specific linear change.

Because ILRs are not directly interpretable, we illustrated model results by back-transforming estimated ILR values into estimates of time spent in each movement behavior using standard procedures.^21^ These estimates were generated for values of cognitive change corresponding to the 25th, 50th, and 75th percentiles of the distribution (referred to as less favorable, median, and more favorable cognitive trajectories, respectively), while marginalizing over the observed distribution of covariates. Using these time-use estimates, we also calculated differences in time use between less favorable and other trajectories. CIs around the time use estimates and differences were derived via nonparametric bootstrapping with 1000 replications; such CIs can be used to indicate precision of estimates but are not related to significance testing. Finally, to assess the statistical significance of the association between cognitive change and the overall movement behavior composition, we used multivariate analysis of covariance with Pillai trace across the 3 ILR outcomes. All analyses were performed in R version 4.4.2 (The R Project for Statistical Computing), with a 2-sided *P* < .05 considered statistically significant.

For additional analyses, we first examined whether results differed by age or sex. We then repeated analyses first including nonlinear time terms in stage-1 models; second, adjusting for self-reported sleep duration at baseline in stage-2 models; and third, adjusting for baseline mobility limitations as a proxy for physical functioning in stage-2 models. Details of additional analyses are provided in eMethods 5 in Supplement 1.

## Results

### Participant Characteristics

The analytic sample included 2529 ELSA participants (1394 [55.1%] female, 1135 [44.9%] male) with a mean (SD) age at the analytic baseline of 56.1 (5.4) years. Participant characteristics at baseline are presented stratified by individual-specific memory or fluency change (<25th percentile, 25th-75th percentile, ≥75th percentile) in Table 1 and Table 2.

**Table 1.** Characteristics of the Analytic Sample at First Cognitive Assessment by Memory Change Percentile (N = 2529)

Characteristic	Memory change percentile, No. (%)^a^			*P* value
	<25th (Less favorable trajectory) (n = 632)	25th-75th (Intermediate trajectory) (n = 1264)	≥75th (More favorable trajectory) (n = 633)	
Age, mean (SD), y	57.9 (6.0)	56.1 (5.3)	54.4 (4.4)	<.001
Sex				
Female	301 (47.6)	675 (53.4)	418 (66.0)	<.001
Male	331 (52.4)	589 (46.6)	215 (34.0)	
Educational attainment				
Less than high school	210 (33.2)	233 (18.4)	59 (9.3)	<.001
High school	339 (53.6)	692 (54.7)	335 (52.9)	
Above high school	83 (13.1)	339 (26.8)	239 (37.8)	
Standardized wealth, mean (SD)	−0.16 (0.65)	−0.01 (1.02)	−0.02 (0.72)	.005
Current smoker	86 (13.6)	154 (12.2)	72 (11.4)	.47
Consumes alcohol	586 (92.7)	1179 (93.3)	598 (94.5)	.43
MVPA				
More than weekly	466 (73.7)	948 (75.0)	496 (78.4)	.02
Weekly	75 (11.9)	167 (13.2)	85 (13.4)	
Less than weekly	91 (14.4)	149 (11.8)	52 (8.2)	
Self-rated hearing				
Excellent	150 (23.7)	351 (27.8)	203 (32.1)	<.001
Very good	185 (29.3)	341 (27.0)	194 (30.6)	
Good	184 (29.1)	396 (31.3)	174 (27.5)	
Fair	88 (13.9)	155 (12.3)	57 (9.0)	
Poor	25 (4.0)	21 (1.7)	5 (0.8)	
CES-D score, mean (SD)	1.5 (1.9)	1.1 (1.7)	1.2 (1.8)	.005
Diagnosis of				
High blood pressure	165 (26.1)	278 (22.0)	122 (19.3)	.01
Diabetes	38 (6.0)	56 (4.4)	12 (1.9)	.001
Cancer	26 (4.1)	45 (3.6)	26 (4.1)	.77
Heart disease	55 (8.7)	93 (7.4)	52 (8.2)	.56
Stroke	3 (0.5)	10 (0.8)	1 (0.2)	.21
Psychiatric conditions	46 (7.3)	92 (7.3)	47 (7.4)	.99

Abbreviations: CES-D, Center for Epidemiologic Studies Depression; MVPA, moderate to vigorous physical activity.

^a^ Cutoffs for memory change correspond to the IQR (−0.011 to 0.004 SD per year).

**Table 2.** Characteristics of the Analytic Sample at First Cognitive Assessment by Fluency Change Percentile (N = 2529)

Characteristic	Fluency change percentile, No. (%)^a^			*P* value
	<25th (Less favorable trajectory) (n = 632)	25th-75th (Intermediate trajectory) (n = 1264)	≥75th (More favorable trajectory) (n = 633)	
Age, mean (SD), y	57.5 (6.0)	56.0 (5.4)	54.9 (4.6)	<.001
Sex				
Female	334 (52.8)	708 (56.0)	352 (55.6)	.41
Male	298 (47.2)	556 (44.0)	281 (44.4)	
Educational attainment				
Less than high school	170 (26.9)	250 (19.8)	82 (13.0)	<.001
High school	340 (53.8)	685 (54.2)	341 (53.9)	
Above high school	122 (19.3)	329 (26.0)	210 (33.2)	
Standardized wealth, mean (SD)	−0.05 (1.17)	−0.07 (0.70)	0.00 (0.83)	.31
Current smoker	69 (10.9)	162 (12.8)	81 (12.8)	.46
Consumes alcohol	577 (91.3)	1192 (94.3)	594 (93.8)	.04
MVPA				
More than weekly	452 (71.5)	943 (74.6)	515 (81.4)	<.001
Weekly	77 (12.2)	182 (14.4)	68 (10.7)	
Less than weekly	103 (16.3)	139 (11.0)	50 (7.9)	
Self-rated hearing				
Excellent	175 (27.7)	344 (27.2)	185 (29.2)	.07
Very good	162 (25.6)	375 (29.7)	183 (28.9)	
Good	198 (31.3)	371 (29.4)	185 (29.2)	
Fair	75 (11.9)	157 (12.4)	68 (10.7)	
Poor	22 (3.5)	17 (1.3)	12 (1.9)	
CES-D score, mean (SD)	1.5 (2.0)	1.2 (1.7)	1.1 (1.6)	.001
Diagnosis of				
High blood pressure	172 (27.2)	272 (21.5)	121 (19.1)	.002
Diabetes	37 (5.9)	49 (3.9)	20 (3.2)	.04
Cancer	20 (3.2)	57 (4.5)	20 (3.2)	.21
Heart disease	52 (8.2)	109 (8.6)	39 (6.2)	.16
Stroke	4 (0.6)	6 (0.5)	4 (0.6)	.87
Psychiatric conditions	57 (9.0)	85 (6.7)	43 (6.8)	.16

Abbreviations: CES-D, Center for Epidemiologic Studies Depression; MVPA, moderate to vigorous physical activity.

^a^ Cutoffs for fluency change correspond to the IQR (0.000 to 0.021 SD per year).

Compared with ELSA participants with cognitive data collected at least once during waves 1 through 9 who were excluded from the analytic sample (eg, who exited the study or died before wave 10 or did not participate in the accelerometer substudy), included participants were similar with respect to sex (1394 of 2529 [55.1%] vs 8619 of 15 872 [54.3%] female; *P* = .46) but younger at baseline (mean [SD] 56.1 [5.4] years vs 62.5 [10.3] years; *P* < .001), of higher socioeconomic position (eg, educated above high school level: 660 of 2529 [26.1%] vs 2252 of 15 872 [14.2%]; *P* < .001; mean [SD] standardized wealth: 0.13 [1.2] vs −0.02 [1.1]; *P* < .001), and generally had a more favorable health behavior and chronic disease profile (eTable 1 in Supplement 1). Compared with the 3622 wave 10 participants who did not participate in the accelerometer substudy, the 3860 substudy participants were similar with respect to age (mean [SD] 68.3 [9.3] years vs 68.2 [10.4] years; *P* = .62) and sex (2143 [55.5%] vs 1978 [54.6%] female; *P* = .44), and were broadly similar across chronic conditions but were of slightly higher socioeconomic position (eg, 1120 [29.0%] vs 971 [26.9%] educated above high school level; *P* < .001), and had a more favorable health behavior profile (eTable 2 in Supplement 1).

The median follow-up duration of the cognitive assessment period was 12 (IQR, 8-16) years, with a maximum follow-up period of 17 years (eFigure 1 in Supplement 1). The mean (SD) age at wave 10 when movement behaviors were assessed was 70.8 (8.7) years. At wave 10, participants spent a mean (SD) of 29 (32) minutes in MVPA, 4.2 (1.1) hours in LPA, 11.3 (2.2) hours in SB, and 7.9 (1.3) hours in sleep per day.

### Cognitive Trajectories and 24-Hour Movement Behavior Composition

The median individual-specific cognitive change was −0.004 (IQR, −0.011 to 0.004) SD per year for memory and 0.010 (IQR, 0.000 to 0.021) SD per year for fluency (eFigure 2 in Supplement 1). These median and IQR values were used to define the median trajectory and the less favorable and more favorable trajectories, respectively. Illustrative trajectories at these values are shown in eFigure 3 in Supplement 1. The median and less favorable memory trajectories declined over time; the more favorable memory trajectory slightly improved. For fluency, the less favorable trajectory was stable over time, whereas the median and more favorable trajectories improved.

Time-use patterns for more favorable, median, and less favorable memory and fluency trajectories are presented in Table 3. After adjustment for covariates, both individual-specific memory and fluency change were associated with the overall movement behavior composition (eTable 3 in Supplement 1). Individuals with faster memory decline engaged in less MVPA and LPA, and more SB and sleep time (Table 3, Table 4). For example, the more favorable trajectory was associated with a mean of 2 (95% CI, 1 to 3) more minutes of MVPA, 14 (95% CI, 8 to 21) more minutes of LPA, 12 (95% CI, –20 to –5) fewer minutes of SB, and 4 (95% CI, –10 to 1) fewer minutes of sleep time compared with the less favorable trajectory.

**Table 3.** Model-Estimated Mean Time Spent in Movement Behaviors and Sleep Time

Cognitive trajectory	Time spent in hours:minutes (95% CI)^a^			
	MVPA	LPA	SB	Sleep
Memory				
Less favorable (−0.011 SD/y)	00:11 (00:10 to 00:12)	03:50 (03:43 to 03:56)	11:45 (11:38 to 11:52)	08:14 (08:10 to 08:19)
Median (−0.004 SD/y)	00:12 (00:11 to 00:13)	03:57 (03:52 to 04:02)	11:39 (11:34 to 11:44)	08:12 (08:09 to 08:16)
More favorable (0.004 SD/y)	00:13 (00:12 to 00:14)	04:04 (03:59 to 04:10)	11:33 (11:27 to 11:40)	08:10 (08:05 to 08:14)
Fluency				
Less favorable (0.000 SD/y)	00:11 (00:10 to 00:12)	03:55 (03:50 to 04:01)	11:40 (11:34 to 11:46)	08:14 (08:10 to 08:18)
Median (0.010 SD/y)	00:12 (00:11 to 00:13)	03:57 (03:51 to 04:01)	11:39 (11:34 to 11:45)	08:12 (08:09 to 08:16)
More favorable (0.021 SD/y)	00:13 (00:12 to 00:13)	03:58 (03:52 to 04:04)	11:39 (11:33 to 11:45)	08:11 (08:06 to 08:15)

Abbreviations: LPA, light physical activity; MVPA, moderate to vigorous physical activity; SB, sedentary behavior.

^a^ Mean time use patterns estimated for values corresponding to the 25th (less favorable), 50th (median), and 75th (more favorable) percentiles of the cognitive change distribution. Time use may not sum exactly to 24 hours due to rounding to the nearest minute. Estimated times are back-transformed from compositional models and therefore represent geometric means of time use. Estimates are derived from compositional models adjusted for age at wave 10 (2021 to 2023), and the following baseline covariates: memory or fluency performance, sex, educational attainment, wealth, high blood pressure, diabetes, cancer, heart disease, stroke, psychiatric conditions, self-rated hearing, alcohol use, smoking status, self-reported physical activity, and depressive symptoms.

**Table 4.** Model-Estimated Differences in Daily Movement Behavior Across Levels of Cognitive Change

Cognitive trajectory	Estimated differences (95% CI), min/d^a^			
	MVPA	LPA	SB	Sleep
Memory				
Less favorable (−0.011 SD/y)	[Reference]	[Reference]	[Reference]	[Reference]
Median (−0.004 SD/y)	1 (1 to 2)	7 (4 to 11)	−6 (−10 to −2)	−2 (−5 to 0)
More favorable (0.004 SD/y)	2 (1 to 3)	14 (8 to 21)	−12 (−20 to −5)	−4 (−10 to 1)
Fluency				
Less favorable (0.000 SD/y)	[Reference]	[Reference]	[Reference]	[Reference]
Median (0.010 SD/y)	1 (0 to 1)	1 (−2 to 4)	−1 (−4 to 2)	−1 (−4 to 1)
More favorable (0.021 SD/y)	2 (1 to 2)	3 (−4 to 8)	−1 (−8 to 5)	−3 (−7 to 2)

Abbreviations: LPA, light physical activity; MVPA, moderate to vigorous physical activity; SB, sedentary behavior.

^a^ Estimated mean differences (95% CI) in minutes per day spent in each movement behavior, relative to the reference value for cognitive change. Positive values indicate more time in the given behavior compared with the reference and negative values indicate less. Estimates are derived from compositional models adjusted for age at wave 10 (2021 to 2023), and the following baseline covariates: memory or fluency performance, sex, educational attainment, wealth, high blood pressure, diabetes, cancer, heart disease, stroke, psychiatric conditions, self-rated hearing, alcohol use, smoking status, self-reported physical activity, and depressive symptoms.

Fluency showed similar patterns to memory (Table 3, Table 4). However, time use differences were smaller than for memory. For example, compared with the less favorable fluency trajectory, the more favorable trajectory was associated with 2 (95% CI, 1 to 2) more minutes of MVPA, 3 (95% CI, –4 to 8) more minutes of LPA, 1 (95% CI, –8 to 5) minute less SB, and 3 (95% CI, –7 to 2) fewer minutes of sleep time.

### Additional Analyses

There was no evidence of effect modification by sex (eTable 4 in Supplement 1), or by age group for fluency (eTable 5 in Supplement 1). However, there was evidence of effect modification by age group for memory (eTable 5, eFigure 4 in Supplement 1): for participants older than 70 years at wave 10, the more favorable memory trajectory was associated with 2 (95% CI, 1 to 3) more minutes of MVPA, 20 (95% CI, 11 to 30) more minutes of LPA, 16 (95% CI, −27 to −5) fewer minutes of SB, and 6 (95% CI, −13 to 1) fewer minutes of sleep time compared with a less favorable trajectory. The corresponding differences for participants 70 years or younger at wave 10 were 2 (95% CI, 0 to 4) more minutes of MVPA, 6 (95% CI, −4 to 15) more minutes of LPA, 6 (95% CI, −16 to 5) fewer minutes of SB, and 2 (95% CI, −11 to 6) fewer minutes of sleep time. Results were substantively unchanged when nonlinear terms were included at the population-level for estimation of individual-specific cognitive change (eTable 6, eFigure 5 in Supplement 1) and when adjusted for baseline self-reported sleep duration (eTable 7, eFigure 6 in Supplement 1) or baseline mobility limitations (eTable 8, eFigure 7 in Supplement 1).

## Discussion

In this compositional analysis of long-term cognitive trajectories and subsequent movement behavior patterns in older adults, we found that less favorable cognitive trajectories were associated with less time being physically active and more sedentary time, independent of cognitive performance and self-reported movement behavior at baseline. Time-use differences were larger for memory than for fluency and for older than for younger participants. Trajectory-related time use differences were largest for LPA and SB, while MVPA and sleep time differences were minor.

Our findings align with evidence suggesting bidirectional associations between cognitive function and movement behaviors,^6,7,8,25,26,27^ with several studies indicating that cognitive performance may exert a stronger influence on physical activity patterns than the reverse.^25,26,27^ They also potentially explain previous observations that declines in activity often begin a decade before dementia diagnosis,^28^ supporting the view that early cognitive changes may contribute to these trends. Null findings in some previous studies^29,30^ may reflect reliance on self-report or a focus on cognitive performance rather than trajectories. We extend this literature by showing that less favorable cognitive trajectories correspond to specific shifts in time use, particularly an increase in sedentary time at the expense of LPA. Possible explanations for these findings include reduced cognitive capacity to plan, initiate, or sustain activity,^26^ alongside co-occurring depressive symptoms, social withdrawal, or early functional limitations that accompany preclinical cognitive decline.^31,32,33,34,35^

In contrast to LPA and SB, memory-related differences in MVPA and sleep time were small. All fluency-related time-use differences were also minor by comparison with memory. MVPA results could reflect the limited time older adults spend in MVPA. The sleep time findings might have occurred because wrist-worn accelerometers primarily capture time in bed, which may be less sensitive to cognitive change than other sleep quality metrics. The weaker findings for fluency may be due to the limited range of fluency decline observed in this analytic sample, which was younger than the broader ELSA cohort; fluency typically declines later than memory.^36^ The larger differences in time use observed for memory compared with fluency are nonetheless broadly consistent with previous findings in older adults.^8^

These findings support the idea that reverse causation may contribute to discrepancies between observational studies and trials, which show modest and inconsistent cognitive benefits of exercise.^37,38^ It was notable that memory-related differences in time use were larger in adults aged older than 70 years, an age range in which cognitive changes may be more detectable and where many observational studies and late-life interventions are conducted. Observational associations in this age range should therefore be interpreted with particular caution. At the same time, the results are consistent with the hypothesis that preserving cognitive health may help support activity patterns in older adulthood, promoting overall well-being and functional independence. Although absolute differences in daily activity time were modest, light activity differences of this magnitude (eg, 10-30 minutes per day) have been associated with lower all-cause mortality in previous observational studies,^39^ underscoring potential clinical relevance.

### Limitations

Residual confounding remains possible. Body mass index was not included because it was measured intermittently in ELSA. However, we adjusted for key related cardiometabolic and behavioral factors. Although ELSA is designed to be nationally representative, the accelerometer and analytic samples were somewhat healthier, more active, and of higher socioeconomic position than the broader ELSA cohort, which may have limited generalizability and biased estimated associations toward the null if less healthy participants with faster cognitive decline were underrepresented. Due to a lack of long-term cognitive data, we could not examine results for other cognitive domains such as executive function, which may also be bidirectionally related to physical activity.^26^ ELSA participants were predominantly White (95%), consistent with the demographic composition of the target population at the time of recruitment^40^; generalizability to more racially and ethnically diverse populations may therefore be limited. The accelerometer wear period occurred during the COVID-19 pandemic, which may limit generalizability of absolute activity levels, although data collection did not coincide with periods of national lockdown. Cognitively stimulating SB may be positively associated with cognitive performance,^41^ but accelerometers cannot make this distinction. Finally, accelerometer-assessed sleep time may be misclassified with sedentary time; this misclassification may be exacerbated in wrist-worn as opposed to thigh worn devices.^42^

## Conclusions

In this cohort study of older adults, a less favorable memory trajectory corresponded to systematically less light physical activity and more sedentary time—equivalent to 1.6 fewer hours of light activity per week overall, and up to 2.3 fewer hours among adults aged older than 70 years. These findings add support to a bidirectional association between cognitive function and physical activity and suggest that the cognitive-to-activity pathway may be more prominent in later life. Observational evidence linking physical activity with cognitive outcomes—as well as dementia prevention strategies and interventions informed by this evidence—should account for the possibility that late-life activity patterns may partly reflect underlying cognitive decline.

## Notes

Supplement 1. **eMethods 1.** Accelerometer data collection and processing **eMethods 2.** Cognitive assessment **eMethods 3.** CES-D Scale **eMethods 4.** Statistical methods **eMethods 5.** Additional analyses **eTable 1.** Comparison of baseline characteristics of ELSA participants aged ≥50 years with at least one wave of cognitive data during waves 1-9 included and excluded from analytic sample **eTable 2.** Comparison of wave 10 ELSA participants aged ≥50 years included and excluded from accelerometry substudy **eTable 3.** Associations between individual-specific cognitive change and movement behavior composition (isometric log-ratios) **eTable 4.** Sex interaction terms **eTable 5.** Age interaction terms **eTable 6.** Associations between individual-specific cognitive change and movement behavior composition (isometric log-ratios) when allowing for nonlinear cognitive change at the population level **eTable 7.** Associations between individual-specific cognitive change and movement behavior composition (isometric log-ratios) additionally adjusted for self-reported sleep duration at baseline **eTable 8.** Associations between individual-specific cognitive change and movement behavior composition (isometric log-ratios) additionally adjusted for mobility limitations at baseline **eFigure 1.** Distribution of waves of follow-up in the analytic sample **eFigure 2.** Distribution of individual-specific memory and fluency change **eFigure 3.** Estimated individual-specific cognitive trajectories at selected percentiles of cognitive change **eFigure 4.** Predicted differences in daily movement behavior across levels of memory change in participants aged ≤70 years (N = 1215) and aged >70 years (N = 1314) at wave 10 **eFigure 5.** Predicted differences in daily movement behavior across levels of cognitive change when allowing for nonlinear cognitive change at the population level (N = 2529) **eFigure 6.** Predicted differences in daily movement behavior across levels of cognitive change additionally adjusted for self-reported sleep duration at baseline (N = 2527) **eFigure 7.** Predicted differences in daily movement behavior across levels of cognitive change additionally adjusted for mobility limitations at baseline (N = 2529)
eReferences

Supplement 2.
Data Sharing Statement
