# Supplementary material for: Cognitive Trajectories and Subsequent Accelerometer-Measured Movement Behavior in Older Adults
Source: JAMA Netw Open. 2026 May 19;9(5):e2613399. doi: 10.1001/jamanetworkopen.2026.13399 (PMC13187880; doi:10.1001/jamanetworkopen.2026.13399)
Supplement: Supplement 1. — eMethods 1. Accelerometer data collection and processing eMethods 2. Cognitive assessment eMethods 3. CES-D Scale eMethods 4. Statistical methods eMethods 5. Additional analyses eTable 1. Comparison of baseline characteristics of ELSA participants aged ≥50 years with at least one wave of cognitive data during waves 1-9 included and excluded from analytic sample eTable 2. Comparison of wave 10 ELSA participants aged ≥50 years included and excluded from accelerometry substudy eTable 3. Associations between individual-specific cognitive change and movement behavior composition (isometric log-ratios) eTable 4. Sex interaction terms eTable 5. Age interaction terms eTable 6. Associations between individual-specific cognitive change and movement behavior composition (isometric log-ratios) when allowing for nonlinear cognitive change at the population level eTable 7. Associations between individual-specific cognitive change and movement behavior composition (isometric log-ratios) additionally adjusted for self-reported sleep duration at baseline eTable 8. Associations between individual-specific cognitive change and movement behavior composition (isometric log-ratios) additionally adjusted for mobility limitations at baseline eFigure 1. Distribution of waves of follow-up in the analytic sample eFigure 2. Distribution of individual-specific memory and fluency change eFigure 3. Estimated individual-specific cognitive trajectories at selected percentiles of cognitive change eFigure 4. Predicted differences in daily movement behavior across levels of memory change in participants aged ≤70 years (N = 1215) and aged >70 years (N = 1314) at wave 10 eFigure 5. Predicted differences in daily movement behavior across levels of cognitive change when allowing for nonlinear cognitive change at the population level (N = 2529) eFigure 6. Predicted differences in daily movement behavior across levels of cognitive change additionally adjusted for self-reported sleep duration at baseline (N [file jamanetwopen-e2613399-s001.pdf]

Supplemental Online Content

Bloomberg M, Brocklebank L, Cavaillès C, Doherty A, Sabia S, Steptoe A. Cognitive trajectories and subsequent accelerometer-measured movement behavior in older adults. *JAMA Netw Open.* 2026;9(5): e2613399. doi: 10.1001/jamanetworkopen.2026.13399

eMethods 1. Accelerometer data collection and processing

eMethods 2. Cognitive assessment

eMethods 3. CES-D Scale

eMethods 4. Statistical methods

eMethods 5. Additional analyses

eTable 1. Comparison of baseline characteristics of ELSA participants aged ≥50 years with at least one wave of cognitive data during waves 1-9 included and excluded from analytic sample

eTable 2. Comparison of wave 10 ELSA participants aged ≥50 years included and excluded from accelerometry substudy

eTable 3. Associations between individual-specific cognitive change and movement behavior composition (isometric log-ratios)

eTable 4. Sex interaction terms

eTable 5. Age interaction terms

eTable 6. Associations between individual-specific cognitive change and movement behavior composition (isometric log-ratios) when allowing for nonlinear cognitive change at the population level

eTable 7. Associations between individual-specific cognitive change and movement behavior composition (isometric log-ratios) additionally adjusted for self-reported sleep duration at baseline

eTable 8. Associations between individual-specific cognitive change and movement behavior composition (isometric log-ratios) additionally adjusted for mobility limitations at baseline

eFigure 1. Distribution of waves of follow-up in the analytic sample

eFigure 2. Distribution of individual-specific memory and fluency change

eFigure 3. Estimated individual-specific cognitive trajectories at selected percentiles of cognitive change

eFigure 4. Predicted differences in daily movement behavior across levels of memory change in participants aged ≤70 years (N=1,215) and aged >70 years (N=1,314) at wave 10

eFigure 5. Predicted differences in daily movement behavior across levels of cognitive change when allowing for nonlinear cognitive change at the population level (N=2,529)

eFigure 6. Predicted differences in daily movement behavior across levels of cognitive change additionally adjusted for self-reported sleep duration at baseline (N=2,527)

eFigure 7. Predicted differences in daily movement behavior across levels of cognitive change additionally adjusted for mobility limitations at baseline (N=2,529)

eReferences

This supplemental material has been provided by the authors to give readers additional information about their work.

eMethods 1. Accelerometer data collection and processing.

Between June 2021 and October 2022, approximately 75% of ELSA households were randomly selected and invited to participate in an accelerometer substudy. Subsequently, to mitigate low response due to COVID-19, all households surveyed between November 2022 and March 2023 were invited.

The Axivity AX3 (Axivity Ltd, Newcastle, UK) is a wrist-worn triaxial accelerometer that has been used in other large-scale cohorts, including the UK Biobank[1] and the China Kadoorie Biobank.[2] Devices were set to capture triaxial acceleration data at a frequency of 100 Hz with a dynamic range of ± 8 gravitational units (*g*). Participants were asked to wear the accelerometer on their dominant wrist 24 hours per day for eight consecutive days. They were informed that they could wear the accelerometer when bathing or swimming but not in extremely high temperature or pressure environments. They were asked to carry on with their normal activities while wearing the accelerometer and did not receive feedback on activity levels until after the device was returned.

For each participant, the average time spent in 24-hour movement behaviors—i.e., time spent in moderate-to-vigorous physical activity, light physical activity, sedentary behavior, and sleep—during the wear period was derived using the Biobank Accelerometer Analysis Tool (https://github.com/OxWearables/biobankAccelerometerAnalysis, v7.1.1), which was developed and validated by the Oxford Wearables Group.[3] Data processing was done in line with previous large scale studies including the UK Biobank.[1] To be included in the analysis, participants had to have at least three days of data and data within each one-hour period of the 24-hour cycle. Non-wear time is defined as uninterrupted periods of at least 60 minutes during which the standard deviation (SD) of acceleration on each axis was less than 13 m*g*. Participants were also excluded if the data could not be parsed, the device could not be calibrated, more than 1% of readings were 'clipped' (i.e., fell outside ±8 *g*) before or after calibration, or the average acceleration was implausibly high (>100 m*g*).

© 2026 Bloomberg M et al. *JAMA Network Open*.

eMethods 2. Cognitive assessment.

The cognitive domains included in the present analysis were episodic memory and verbal fluency. These domains are important for day-to-day function and show decline with aging and dementia.[4] Of the three cognitive domains assessed in ELSA and administered at enough waves to produce long-term cognitive trajectories (memory, fluency, and orientation in time), memory and fluency were chosen because their assessments are less susceptible to ceiling effects. Cognitive trajectories were produced using cognitive testing from waves 1-9 (2002-03 to 2018-19).

Memory was assessed using immediate and delayed recall tasks, in which participants were read a 10-word list and asked to recall it immediately and then after a short delay. These scores were summed to give a summary recall score (range: 0-20). Fluency was assessed using the animal naming task,[5] where participants were asked to name out loud as many animals as possible in one minute. Memory was assessed at every wave and fluency at all waves except wave 6. To facilitate interpretation, cognitive scores were standardized to the analytic sample.

© 2026 Bloomberg M et al. *JAMA Network Open*.

eMethods 3. CES-D Scale.

Depressive symptoms were assessed using the 8-item Center for Epidemiologic Studies Depression (CES-D) Scale,[6] which requires participants to report whether they often (yes or no): 1) feel depressed; 2) feel everything is an effort; 3) have restless sleep; 4) are happy; 5) feel lonely; 6) feel sad; 7) cannot get going; and 8) enjoy life. One point was scored for each item with ‘happy’ and ‘enjoyed life’ reverse scored. A higher score indicates more depressive symptoms (range: 0-8).

© 2026 Bloomberg M et al. *JAMA Network Open*.

eMethods 4. Statistical methods.

We used a two-stage modeling approach to determine whether cognitive trajectories were associated with subsequent movement behavior patterns. This involved first estimating individual-specific cognitive change per year using linear mixed models, then using this estimated cognitive change as a predictor in models with the composition of movement behaviors as the outcome. In the context of cognitive aging, using linear mixed models is often preferable to other commonly-used modeling techniques such as group-based trajectory models, which produce multiple parallel trajectories (a "rainbow effect"[7]) when interindividual variability in the intercept (i.e., where cognitive performance starts out) is much higher than that of the slope (i.e., the rate of cognitive decline). This precludes examining differences in cognitive decline. Our modeling approach allowed us to use all available data and robustly handle missing data and differences in follow-up duration, assuming data missing at random.[8] Similar two-stage approaches using linear mixed models have been applied in previous studies of physical activity and cognitive health.[9,10]

In stage one, we produced individual-specific linear slopes as a summary measure of cognitive decline, derived from linear mixed models fitted separately for memory and fluency. We used a backwards time scale (i.e., years prior to the accelerometer assessment) so that time zero corresponds to the measurement of movement behaviors. This allows clearer interpretation of the timing between cognitive change and subsequent activity patterns and ensures the interval between exposures (cognitive assessments) and outcome (accelerometer data) is consistently defined across participants. The models included a random intercept and a random slope for the linear time term at the individual level (with an unstructured covariance matrix) to capture interindividual variability, and time as a fixed effect. We did not include additional covariates in the first-stage models, as our goal was to produce a summary measure of each participant's cognitive decline over time, rather than decline adjusted for demographic or health-related factors. This approach allowed all sources of interindividual variability in linear cognitive decline—including those attributable to sociodemographic characteristics or underlying health status—to be retained in the individual-specific slope estimates.

Individual-specific slope was computed by summing the fixed-effect coefficient for time and the individual's random effect on time. This value reflects the participant's estimated rate of cognitive change per year. More negative values indicate more rapid cognitive decline. We plotted predicted cognitive trajectories at the 25th, 50th, and 75th percentiles of individual-specific memory and fluency change to illustrate differences in slope across the distribution of cognitive change.

In the second stage, we applied CoDA to examine associations between memory or fluency decline and the 24-hour composition of movement behaviors (MVPA, LPA, SB, and sleep) measured at wave 10. CoDA is a well-established method for analyzing compositional data, where the parts sum to a whole (e.g., 24 hours in a day).[11-14] It converts the proportions of time spent in each behavior into isometric log-ratio (ILR) coordinates, which express the relative balance of one behavior compared to others in a mathematically appropriate way for compositional data. This transformation avoids statistical issues that arise when analyzing raw proportions directly due to their inherent interdependence. While ILRs themselves are not interpretable, they allow us to perform appropriate regression analysis on compositional data, after which results can be translated back into meaningful time-use differences. As compositional data cannot include zeros, we used multiplicative replacement to impute small values (3.6 seconds) for zeros, as has been done previously.[15]

We used the *ilr* function from the *compositions* package[12] in R to convert time-use data into ILR coordinates. This function constructs ILRs by sequentially comparing one behavior to the remaining set. We defined three ILRs: 1) MVPA relative to LPA, SB, and sleep; 2) LPA relative to SB and sleep; and 3) SB relative to sleep. Each ILR coordinate was then used as the dependent variable in a separate linear regression model, with individual-specific linear memory or fluency change as the predictor.

All second-stage models were adjusted for age at wave 10, sex, and the following baseline covariates: educational attainment, wealth, smoking status, alcohol consumption, self-reported physical activity level, self-rated hearing, depressive symptoms, high blood pressure, heart disease, stroke, cancer, diabetes, and psychiatric conditions. Covariates were selected to adjust for potential confounding; variables plausibly lying on the causal pathway between cognitive decline and movement behaviors were not included to avoid over-adjustment. Because missingness was minor (<5%) for all covariates, missing covariates were singly imputed using predictive mean matching,[16] with imputation models including all other covariates and the primary exposure and outcome variables. We also adjusted for memory or fluency performance at baseline to account for differences in baseline performance, after determining that in fully adjusted models there was no evidence of interaction between performance at baseline and individual-specific linear change.

© 2026 Bloomberg M et al. *JAMA Network Open*.

To assess whether cognitive decline was associated with the overall movement behavior composition, we used multivariate analysis of covariance (MANCOVA) with Pillai's Trace across the three ILR outcomes. Because ILRs are not directly interpretable, we illustrated model results by back-transforming predicted ILR values into estimates of time spent in each movement behavior using standard procedures.[12] These predictions were generated for values of cognitive change corresponding to the 25th, 50th, and 75th percentiles of the distribution (referred to as 'less favorable', 'median', and 'more favorable' cognitive trajectories, respectively). We also calculated the model-predicted differences in time use between less favorable and other trajectories. Confidence intervals around the predicted time use estimates were derived via nonparametric bootstrapping with 1000 replications; such confidence intervals can be used to indicate precision of estimates but are not related to significance testing. All analyses were performed in R version 4.4.2, with two-sided p-values<0.05 considered statistically significant.

© 2026 Bloomberg M et al. *JAMA Network Open*.

eMethods 5. Additional analyses.

First, to determine whether there were differences in results by age or sex, we checked for evidence of interaction between cognitive change and age group at wave 10 (≤70 or >70 years, to correspond to the median age at wave 10) or sex (male or female) using MANCOVA with Pillai's trace to assess statistical significance of interaction terms across the ILR outcomes. This led us to rerun stage-two memory models stratified by age group at wave 10.

Second, as cognitive performance often changes non-linearly with age, we repeated the analysis including nonlinear time terms (quadratic and cubic terms) as fixed effects in stage-one models. This allowed us to determine whether excluding nonlinear terms changed how participants were ranked according to their estimated cognitive change. In these models, the estimated individual-specific change no longer reflects an absolute rate of change per year but instead indicates the extent to which a participant's trajectory curves more steeply downward.

Third, as self-reported sleep duration was reported at waves 4, 6, and 8 of ELSA only, we repeated analyses with the cognitive assessment period restricted to waves 4-9, to be able to adjust for baseline self-reported sleep duration in stage-two models.

Finally, we checked whether adjustment for mobility limitations (as a proxy for physical functioning) at baseline affected the results. Participants were asked to report whether they had difficulty with each of the following (yes or no): walking 100 yards, sitting down for two hours, getting up from a chair, climbing a flight of stairs, stooping/kneeling/crouching, lifting/carrying 10 pounds, picking up a 5p coin, reaching/extending the arms, or pulling/pushing large objects. Participants were given one point for each item they reported difficulty with to give a mobility limitation index (range: 0-9), which was included as a continuous variable in stage-two models.

© 2026 Bloomberg M et al. *JAMA Network Open*.

eTable 1. Comparison of baseline characteristics of ELSA participants aged ≥50 years with at least one wave of cognitive data during waves 1-9 included and excluded from analytic sample.

	In analytic sample: No (N=15,872)	In analytic sample: Yes (N=2,529)	*P-value*
Age, mean (SD)	62.5 (10.3)	56.1 (5.4)	<0.001
Sex			
Male	7253 (45.7)	1135 (44.9)	
Female	8619 (54.3)	1394 (55.1)	0.46
Educational attainment			
Less than high school	6888 (43.4)	502 (19.8)	
High school	6732 (42.4)	1367 (54.1)	<0.001
Above high school	2252 (14.2)	660 (26.1)	
Standardized wealth, mean (SD)	-0.02 (1.1)	0.13 (1.2)	<0.001
Current smoker	2986 (18.8)	311 (12.3)	<0.001
Consumes alcohol	13099 (88.4)	2361 (93.4)	<0.001
MVPA			
More than weekly	9773 (61.6)	1910 (75.5)	
Weekly	2258 (14.2)	327 (12.9)	<0.001
Less than weekly	3837 (24.2)	292 (11.5)	
Self-rated hearing			
Excellent	3749 (23.6)	704 (27.8)	
Very good	4273 (26.9)	720 (28.5)	<0.001
Good	4724 (29.8)	754 (29.8)	
Fair	2395 (15.1)	300 (11.9)	
Poor	726 (4.6)	51 (2.0)	
CES-D score, mean (SD)	1.6 (2.0)	1.2 (1.8)	<0.001
Diagnosis of:			
High blood pressure	5248 (33.1)	565 (22.3)	<0.001
Diabetes	1153 (7.3)	106 (4.2)	<0.001
Cancer	912 (5.7)	97 (3.8)	<0.001
Heart disease	2365 (14.9)	165 (6.5)	<0.001
Stroke	532 (3.4)	14 (0.6)	<0.001
Psychiatric conditions	1166 (7.3)	185 (7.3)	0.98

Data shown are N (%) unless otherwise indicated.
Abbreviations: SD, standard deviation; IQR, interquartile range; MVPA, moderate-to-vigorous physical activity; CES-D, Center for Epidemiologic Studies Depression.

© 2026 Bloomberg M et al. *JAMA Network Open*.

eTable 2. Comparison of wave 10 ELSA participants aged ≥50 years included and excluded from accelerometry substudy.

	In accelerometry substudy: No (N=3,622)	Yes (N=3,860)	*P-value*
Age, mean (SD)	68.2 (10.4)	68.3 (9.3)	0.62
Sex			
Male	1644 (45.4)	1717 (44.5)	
Female	1978 (54.6)	2143 (55.5)	0.44
Educational attainment			
Less than high school	823 (22.8)	686 (17.8)	
High school	1819 (50.3)	2052 (53.2)	<0.001
Above high school	971 (26.9)	1120 (29.0)	
Standardized wealth, mean (SD)	-0.05 (0.94)	0.04 (1.1)	<0.001
Current smoker	335 (9.3)	261 (6.8)	<0.001
Consumes alcohol	2699 (84.2)	3279 (86.7)	0.003
MVPA			
More than weekly	2207 (61.0)	2674 (69.3)	
Weekly	459 (12.7)	456 (11.8)	<0.001
Less than weekly	954 (26.4)	730 (18.9)	
Self-rated hearing			
Excellent	682 (18.8)	689 (17.9)	
Very good	988 (27.3)	1113 (28.8)	0.002
Good	1150 (31.8)	1238 (32.1)	
Fair	589 (16.3)	665 (17.2)	
Poor	212 (5.9)	154 (4.0)	
CES-D score, mean (SD)	1.5 (1.9)	1.4 (1.8)	<0.001
Diagnosis of:			
High blood pressure	1609 (44.4)	1771 (45.9)	0.22
Diabetes	491 (13.6)	480 (12.4)	0.16
Cancer	555 (15.3)	639 (16.6)	0.16
Heart disease	933 (25.8)	1015 (26.3)	0.62
Stroke	230 (6.4)	181 (4.7)	0.002
Psychiatric conditions	604 (16.7)	640 (16.6)	0.92

Data shown are N (%) unless otherwise indicated.
Abbreviations: SD, standard deviation; IQR, interquartile range; MVPA, moderate-to-vigorous physical activity; CES-D, Center for Epidemiologic Studies Depression.

© 2026 Bloomberg M et al. *JAMA Network Open*.

eTable 3. Associations between individual-specific cognitive change and movement behavior composition (isometric log-ratios).

	Coefficient (95% CI)	P-value
**Memory**		
ILR 1: MVPA relative to LPA, SB, and sleep	-5.81 (-9.98 to -1.65)	0.006
ILR 2: LPA relative to SB and sleep	-7.66 (-10.86 to -4.47)	<0.001
ILR 3: SB relative to sleep	-4.91 (-7.00 to -2.82)	<0.001
Overall p-value*	<0.001	
**Fluency**		
ILR 1: MVPA relative to LPA, SB, and sleep	-4.09 (-6.59 to -1.58)	0.001
ILR 2: LPA relative to SB and sleep	-2.85 (-4.78 to -0.93)	0.004
ILR 3: SB relative to sleep	-2.19 (-3.45 to -0.93)	<0.001
Overall p-value*	<0.001	

ILRs express the relative balance of one behavior compared to the others. Coefficients show change in specified ILR per standard deviation change in individual-specific cognitive slope. Coefficients are not directly interpretable and presented for completeness. Models are adjusted for age at wave 10 (2021-23), sex, and the following baseline covariates: memory or fluency performance, educational attainment, wealth, high blood pressure, diabetes, cancer, heart disease, stroke, psychiatric conditions, self-rated hearing, alcohol consumption, smoking status, self-reported physical activity, and depressive symptoms.
*P-values for the overall association between cognitive decline and the movement behavior composition are from multivariate analysis of covariance (MANCOVA), using Pillai's Trace.
Abbreviations: CI, confidence interval; ILR, isometric log-ratio; MVPA, moderate-to-vigorous physical activity; LPA, light physical activity; SB, sedentary behavior.

© 2026 Bloomberg M et al. *JAMA Network Open*.

eTable 4. Sex interaction terms.

	Coefficient (95% CI)	P-value
**Memory change x sex**		
ILR 1: MVPA relative to LPA, SB, and sleep	-5.58 (-13.26, 2.10)	0.15
ILR 2: LPA relative to SB and sleep	-3.91 (-9.79, 1.98)	0.19
ILR 3: SB relative to sleep	-3.74 (-7.59, 0.11)	0.06
Overall p-value*	0.21	
**Fluency change x sex**		
ILR 1: MVPA relative to LPA, SB, and sleep	0.64 (-4.24, 5.52)	0.80
ILR 2: LPA relative to SB and sleep	-0.37 (-4.12, 3.38)	0.85
ILR 3: SB relative to sleep	-0.59 (-3.04, 1.87)	0.64
Overall p-value*	0.75	

ILRs express the relative balance of one behavior compared to the others. Coefficients are for the interaction of memory or fluency change with sex (male or female; reference: male). Coefficients are not directly interpretable and presented for completeness. Models are adjusted for age at wave 10 (2021-23), sex, and the following baseline covariates: memory or fluency performance, educational attainment, wealth, high blood pressure, diabetes, cancer, heart disease, stroke, psychiatric conditions, self-rated hearing, alcohol consumption, smoking status, self-reported physical activity, and depressive symptoms.
*P-values for the overall significance of the interaction terms are from multivariate analysis of covariance (MANCOVA), using Pillai's Trace.
Abbreviations: CI, confidence interval; ILR, isometric log-ratio; MVPA, moderate-to-vigorous physical activity; LPA, light physical activity; SB, sedentary behavior.

© 2026 Bloomberg M et al. *JAMA Network Open*.

eTable 5. Age interaction terms.

	Coefficient (95% CI)	P-value
**Memory change x age group**		
ILR 1: MVPA relative to LPA, SB, and sleep	-4.69 (-12.91, 3.53)	0.26
ILR 2: LPA relative to SB and sleep	-8.96 (-15.25, -2.66)	0.005
ILR 3: SB relative to sleep	-5.62 (-9.74, -1.50)	0.008
Overall p-value*	0.01	
**Fluency change x age group**		
ILR 1: MVPA relative to LPA, SB, and sleep	1.18 (-4.02, 6.37)	0.66
ILR 2: LPA relative to SB and sleep	-0.37 (-4.37, 3.63)	0.86
ILR 3: SB relative to sleep	0.25 (-2.36, 2.86)	0.85
Overall p-value*	0.75	

ILRs express the relative balance of one behavior compared to the others. Coefficients are for interaction of memory or fluency change with age group at wave 10 (≤70 or >70; reference: ≤70). Coefficients are not directly interpretable and presented for completeness. Models are adjusted for sex and the following baseline covariates: memory or fluency performance, educational attainment, wealth, high blood pressure, diabetes, cancer, heart disease, stroke, psychiatric conditions, self-rated hearing, alcohol consumption, smoking status, self-reported physical activity, and depressive symptoms.

*P-values for the overall significance of the interaction terms are from multivariate analysis of covariance (MANCOVA), using Pillai's Trace.

Abbreviations: CI, confidence interval; ILR, isometric log-ratio; MVPA, moderate-to-vigorous physical activity; LPA, light physical activity; SB, sedentary behavior.

© 2026 Bloomberg M et al. *JAMA Network Open*.

eTable 6. Associations between individual-specific cognitive change and movement behavior composition (isometric log-ratios) when allowing for nonlinear cognitive change at the population level.

	Coefficient (95% CI)	P-value
**Memory**		
ILR 1: MVPA relative to LPA, SB, and sleep	-5.38 (-9.31, -1.46)	0.007
ILR 2: LPA relative to SB and sleep	-7.12 (-10.13, -4.11)	<0.001
ILR 3: SB relative to sleep	-4.58 (-6.55, -2.61)	<0.001
Overall p-value*	<0.001	
**Fluency**		
ILR 1: MVPA relative to LPA, SB, and sleep	-4.05 (-6.55, -1.55)	0.001
ILR 2: LPA relative to SB and sleep	-2.82 (-4.74, -0.90)	0.004
ILR 3: SB relative to sleep	-2.17 (-3.43, -0.91)	<0.001
Overall p-value*	<0.001	

ILRs express the relative balance of one behavior compared to the others. Coefficients show change in specified ILR per standard deviation change in individual-specific cognitive slope. Coefficients are not directly interpretable and presented for completeness. Models are adjusted for age at wave 10 (2021-23), sex, and the following baseline covariates: memory or fluency performance, educational attainment, wealth, high blood pressure, diabetes, cancer, heart disease, stroke, psychiatric conditions, self-rated hearing, alcohol consumption, smoking status, self-reported physical activity, and depressive symptoms.
*P-values for the overall association between cognitive decline and the movement behavior composition are from multivariate analysis of covariance (MANCOVA), using Pillai's Trace.
Abbreviations: CI, confidence interval; ILR, isometric log-ratio; MVPA, moderate-to-vigorous physical activity; LPA, light physical activity; SB, sedentary behavior.

© 2026 Bloomberg M et al. *JAMA Network Open*.

eTable 7. Associations between individual-specific cognitive change and movement behavior composition (isometric log-ratios) additionally adjusted for self-reported sleep duration at baseline.

	Coefficient (95% CI)	P-value
**Memory**		
ILR 1: MVPA relative to LPA, SB, and sleep	-4.08 (-7.89, -0.26)	0.03
ILR 2: LPA relative to SB and sleep	-6.27 (-9.18, -3.35)	<0.001
ILR 3: SB relative to sleep	-3.91 (-5.82, -2.01)	<0.001
Overall p-value*	<0.001	
**Fluency**		
ILR 1: MVPA relative to LPA, SB, and sleep	-1.78 (-3.89, 0.32)	0.10
ILR 2: LPA relative to SB and sleep	-1.14 (-2.75, 0.48)	0.17
ILR 3: SB relative to sleep	-1.12 (-2.18, -0.07)	0.04
Overall p-value*	<0.001	

ILRs express the relative balance of one behavior compared to the others. Coefficients show change in specified ILR per standard deviation change in individual-specific cognitive slope. Coefficients are not directly interpretable and presented for completeness. Models are adjusted for age at wave 10 (2021-23), sex, and the following baseline covariates: memory or fluency performance, educational attainment, wealth, high blood pressure, diabetes, cancer, heart disease, stroke, psychiatric conditions, self-rated hearing, alcohol consumption, smoking status, self-reported physical activity, depressive symptoms, and self-reported sleep duration.

*P-values for the overall association between cognitive decline and the movement behavior composition are from multivariate analysis of covariance (MANCOVA), using Pillai's Trace.

Abbreviations: CI, confidence interval; ILR, isometric log-ratio; MVPA, moderate-to-vigorous physical activity; LPA, light physical activity; SB, sedentary behavior.

© 2026 Bloomberg M et al. *JAMA Network Open*.

eTable 8. Associations between individual-specific cognitive change and movement behavior composition (isometric log-ratios) additionally adjusted for mobility limitations at baseline.

	Coefficient (95% CI)	P-value
**Memory**		
ILR 1: MVPA relative to LPA, SB, and sleep	-5.14 (-9.31, -0.97)	0.02
ILR 2: LPA relative to SB and sleep	-6.76 (-9.94, -3.58)	<0.001
ILR 3: SB relative to sleep	-4.49 (-6.58, -2.41)	<0.001
Overall p-value*	<0.001	
**Fluency**		
ILR 1: MVPA relative to LPA, SB, and sleep	-3.90 (-6.40, -1.40)	0.002
ILR 2: LPA relative to SB and sleep	-2.60 (-4.51, -0.69)	0.008
ILR 3: SB relative to sleep	-2.07 (-3.32, -0.81)	0.001
Overall p-value*	<0.001	

ILRs express the relative balance of one behavior compared to the others. Coefficients show change in specified ILR per standard deviation change in individual-specific cognitive slope. Coefficients are not directly interpretable and presented for completeness. Models are adjusted for age at wave 10 (2021-23), sex, and the following baseline covariates: memory or fluency performance, educational attainment, wealth, high blood pressure, diabetes, cancer, heart disease, stroke, psychiatric conditions, self-rated hearing, alcohol consumption, smoking status, self-reported physical activity, depressive symptoms, and number of mobility limitations.

*P-values for the overall association between cognitive decline and the movement behavior composition are from multivariate analysis of covariance (MANCOVA), using Pillai's Trace.

Abbreviations: CI, confidence interval; ILR, isometric log-ratio; MVPA, moderate-to-vigorous physical activity; LPA, light physical activity; SB, sedentary behavior.

© 2026 Bloomberg M et al. *JAMA Network Open*.

eFigure 1. Distribution of waves of follow-up in the analytic sample.

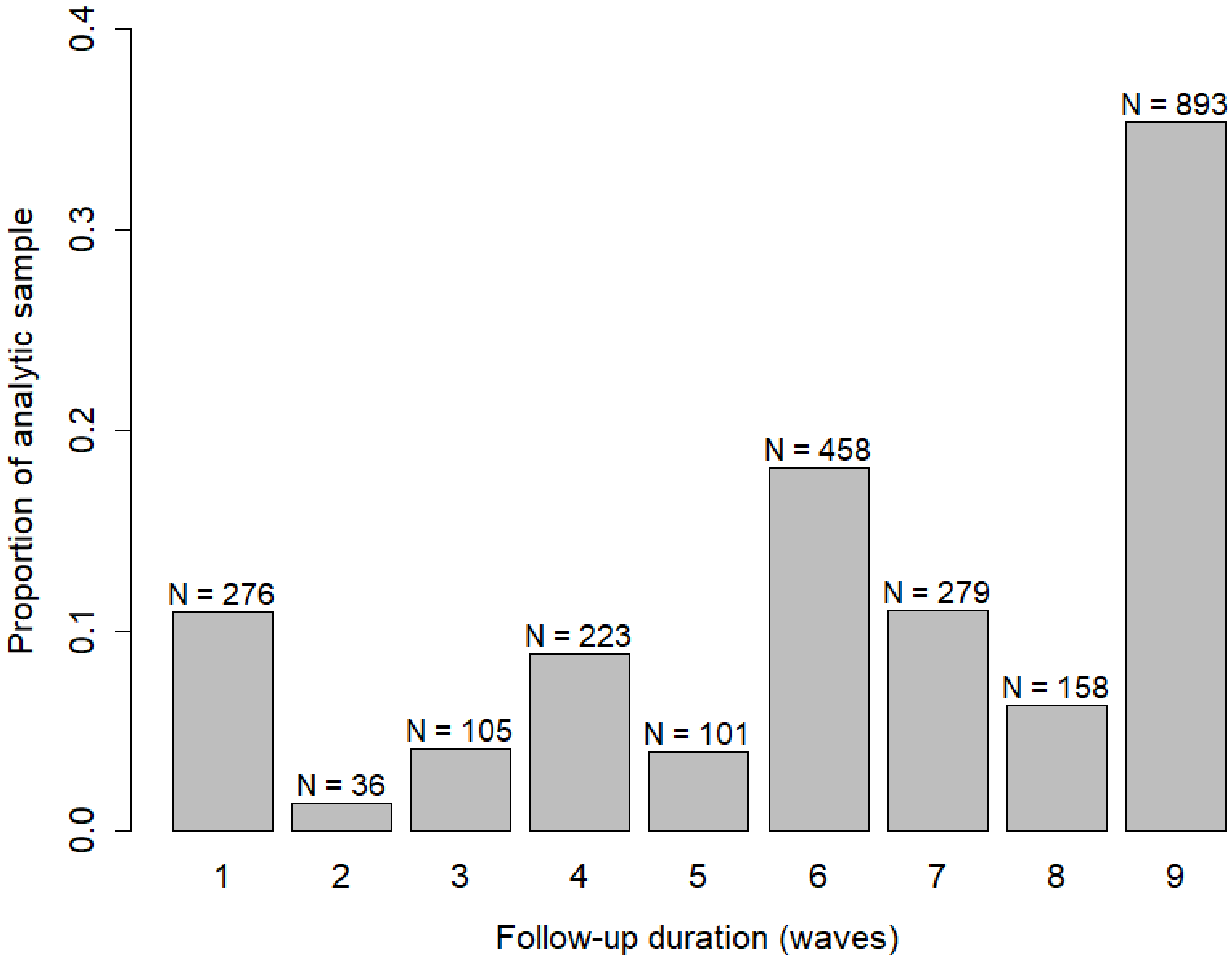

Each wave corresponds to approximately two years (i.e., an individual participating in four waves of data collection has approximately eight years of follow-up).

© 2026 Bloomberg M et al. *JAMA Network Open*.

eFigure 2. Distribution of individual-specific memory and fluency change.

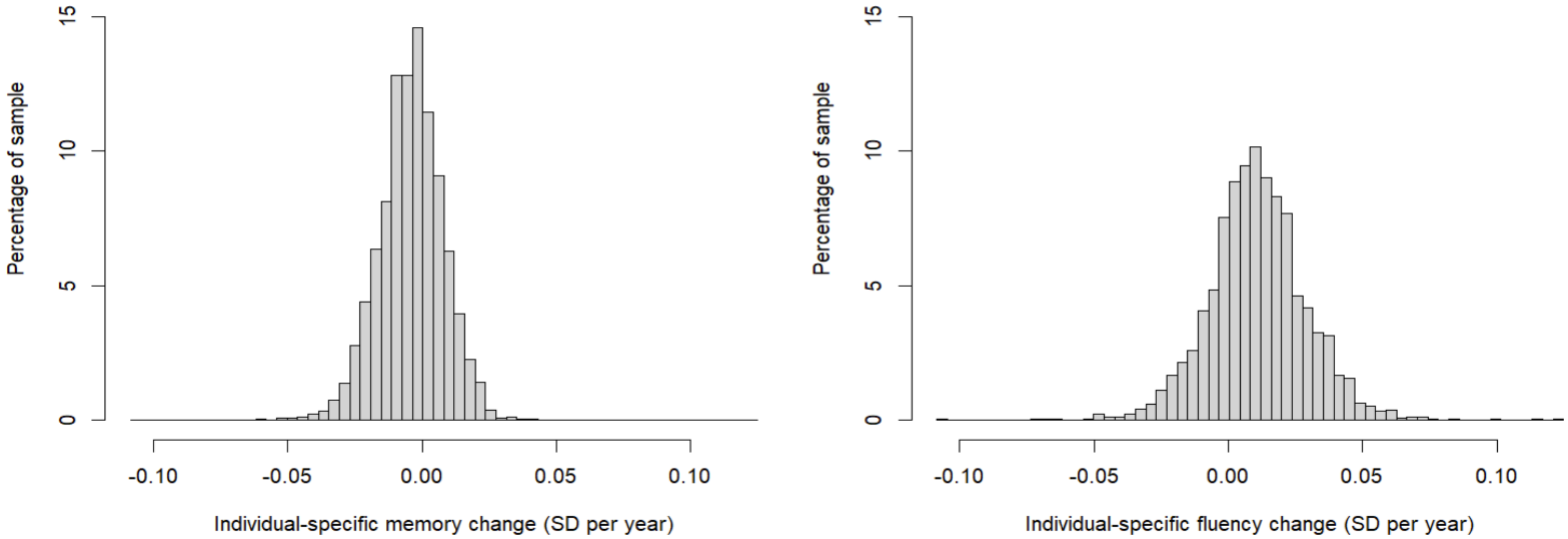

Abbreviations: SD, standard deviation.

© 2026 Bloomberg M et al. *JAMA Network Open*.

eFigure 3. Estimated individual-specific cognitive trajectories at selected percentiles of cognitive change.

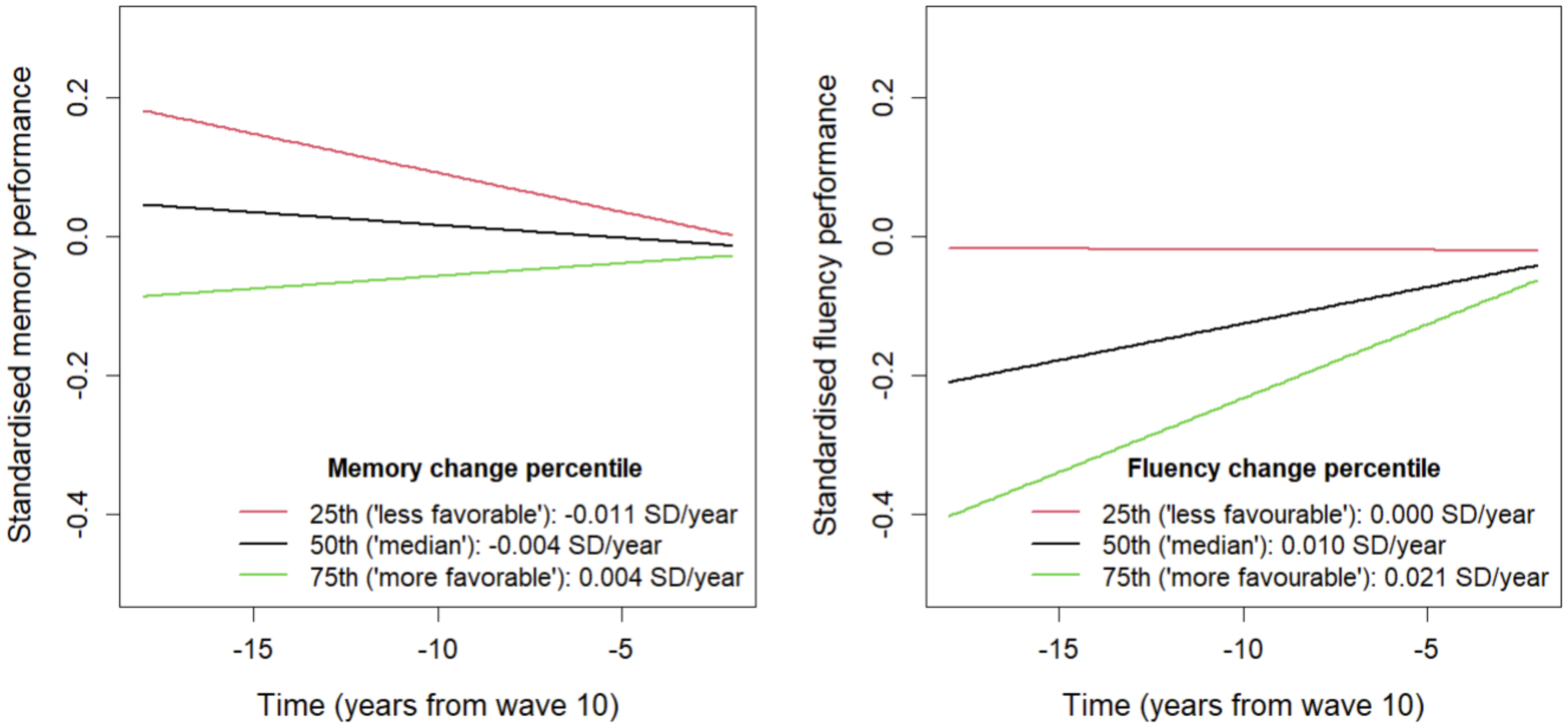

Model-predicted cognitive trajectories for memory and fluency at the 25th ('less favorable'), 50th ('median'), and 75th ('more favorable') percentiles of individual-specific cognitive change. These are illustrative predictions obtained using the fixed-effects portion of the mixed models; they do not represent trajectories of distinct participant subgroups. Trajectories are centered such that cognitive score equals 0 at wave 10 (the time of accelerometer measurement) to highlight differences in slope.

© 2026 Bloomberg M et al. *JAMA Network Open*.

eFigure 4. Predicted differences in daily movement behavior across levels of memory change in participants aged ≤70 years (N=1,215) and aged >70 years (N=1,314) at wave 10.

Aged ≤70 years at wave 10	MVPA	LPA	SB	Sleep
Less favourable	Reference	Reference	Reference	Reference
Median	+1 (0 to +2)	+3 (-2 to +8)	-3 (-8 to +3)	-1 (-5 to +3)
More favourable	+2 (0 to +4)	+6 (-4 to +15)	-6 (-16 to +5)	-2 (-10 to +6)

Aged >70 years at wave 10	MVPA	LPA	SB	Sleep
Less favourable	Reference	Reference	Reference	Reference
Median	+1 (0 to +1)	+10 (+5 to +14)	-8 (-13 to -3)	-3 (-6 to +1)
More favourable	+2 (+1 to +3)	+20 (+11 to +30)	-16 (-27 to -5)	-6 (-13 to +1)

Predicted average differences (95% confidence interval) in minutes per day spent in each movement behavior, relative to the reference value for memory change. Positive values indicate more time in the given behavior compared to the reference and negative values indicate less. Memory change was modelled as a continuous variable, and predictions are shown for values corresponding to the 25th ('less favorable'=-0.011 SD/year), 50th ('median'=-0.004 SD/year), and 75th ('more favorable'=0.004 SD/year) percentiles of the memory change distribution. Estimates are derived from compositional models adjusted for age at wave 10 (2021-23), and the following baseline covariates: memory or fluency performance, sex, educational attainment, wealth, high blood pressure, diabetes, cancer, heart disease, stroke, psychiatric conditions, self-rated hearing, alcohol use, smoking status, self-reported physical activity, and depressive symptoms.
Abbreviations: MVPA, moderate-to-vigorous physical activity; LPA, light physical activity; SB, sedentary behavior.

© 2026 Bloomberg M et al. *JAMA Network Open*.

eFigure 5. Predicted differences in daily movement behavior across levels of cognitive change when allowing for nonlinear cognitive change at the population level (N=2,529).

**Memory trajectory**	MVPA	LPA	SB	Sleep
Less favourable	Reference	Reference	Reference	Reference
Median	+1 (+1 to +2)	+7 (+4 to +11)	-6 (-10 to -2)	-2 (-5 to 0)
More favourable	+2 (+1 to +3)	+15 (+7 to +22)	-12 (-20 to -5)	-4 (-10 to 0)

**Fluency trajectory**	MVPA	LPA	SB	Sleep
Less favourable	Reference	Reference	Reference	Reference
Median	+1 (0 to +1)	+1 (-2 to +4)	-1 (-4 to +3)	-1 (-4 to +1)
More favourable	+2 (+1 to +2)	+3 (-4 to +9)	-1 (-8 to +5)	-3 (-7 to +1)

Predicted average differences (95% confidence interval) in minutes per day spent in each movement behavior, relative to the reference value for cognitive change. Positive values indicate more time in the given behavior compared to the reference and negative values indicate less. Cognitive change was modelled as a continuous variable, and predictions are shown for values corresponding to the 25th ('less favorable': memory=-0.011 SD/year, fluency=0.000 SD/year), 50th ('median': memory=-0.004 SD/year, fluency=0.010 SD/year), and 75th ('more favorable': memory=0.004 SD/year, fluency=0.021 SD/year) percentiles of the cognitive change distribution. Models to produce individual-specific cognitive change include nonlinear time terms ($time^2$ in memory models and $time^3$ in fluency models) as fixed effects. Individual-specific linear change no longer reflects an absolute rate of change per year but instead indicates the extent to which a participant's trajectory curves more steeply downward. Estimates are derived from compositional models adjusted for age at wave 10 (2021-23), and the following baseline covariates: memory or fluency performance, sex, educational attainment, wealth, high blood pressure, diabetes, cancer, heart disease, stroke, psychiatric conditions, self-rated hearing, alcohol use, smoking status, self-reported physical activity, and depressive symptoms.
Abbreviations: MVPA, moderate-to-vigorous physical activity; LPA, light physical activity; SB, sedentary behavior.

© 2026 Bloomberg M et al. *JAMA Network Open*.

eFigure 6. Predicted differences in daily movement behavior across levels of cognitive change additionally adjusted for self-reported sleep duration at baseline (N=2,527).

**Memory trajectory**	MVPA	LPA	SB	Sleep
Less favourable	Reference	Reference	Reference	Reference
Median	+1 (0 to +1)	+7 (+4 to +11)	-6 (-10 to -3)	-2 (-5 to +1)
More favourable	+2 (+1 to +3)	+14 (+7 to +21)	-12 (-19 to -5)	-4 (-8 to +1)

**Fluency trajectory**	MVPA	LPA	SB	Sleep
Less favourable	Reference	Reference	Reference	Reference
Median	0 (0 to +1)	+1 (-3 to +4)	+1 (-3 to +4)	-2 (-4 to 0)
More favourable	+1 (0 to +2)	+1 (-5 to +7)	+2 (-5 to +9)	-4 (-8 to +1)

Predicted average differences (95% confidence interval) in minutes per day spent in each movement behavior, relative to the reference value for cognitive change. Positive values indicate more time in the given behavior compared to the reference and negative values indicate less. Cognitive change was modelled as a continuous variable, and predictions are shown for values corresponding to the 25th (‘less favorable’: memory=-0.011 SD/year, fluency=0.000 SD/year), 50th (‘median’: memory=-0.004 SD/year, fluency=0.010 SD/year), and 75th (‘more favorable’: memory=0.004 SD/year, fluency=0.021 SD/year) percentiles of the cognitive change distribution. Estimates are derived from compositional models adjusted for age at wave 10 (2021-23), and the following baseline covariates: memory or fluency performance, sex, educational attainment, wealth, high blood pressure, diabetes, cancer, heart disease, stroke, psychiatric conditions, self-rated hearing, alcohol use, smoking status, self-reported physical activity, and depressive symptoms. Self-reported sleep duration was assessed at waves 4, 6, and 8; cognitive assessment period is restricted to waves 4-9 and compositional models are additionally adjusted for self-reported sleep duration at baseline.
Abbreviations: MVPA, moderate-to-vigorous physical activity; LPA, light physical activity; SB, sedentary behavior

© 2026 Bloomberg M et al. *JAMA Network Open*.

eFigure 7. Predicted differences in daily movement behavior across levels of cognitive change additionally adjusted for mobility limitations at baseline (N=2,529).

**Memory trajectory**	MVPA	LPA	SB	Sleep
Less favorable	Reference	Reference	Reference	Reference
Median	+1 (0 to +1)	+6 (+3 to +10)	-5 (-9 to -1)	-2 (-5 to 0)
More favorable	+2 (+1 to +3)	+13 (+6 to +20)	-10 (-17 to -2)	-5 (-10 to 0)

**Fluency trajectory**	MVPA	LPA	SB	Sleep
Less favorable	Reference	Reference	Reference	Reference
Median	+1 (0 to +1)	+1 (-2 to +4)	0 (-3 to +3)	-2 (-4 to +1)
More favorable	+1 (+1 to +2)	+2 (-4 to +8)	0 (-7 to +6)	-3 (-8 to +1)

Predicted average differences (95% confidence interval) in minutes per day spent in each movement behavior, relative to the reference value for cognitive change. Positive values indicate more time in the given behavior compared to the reference and negative values indicate less. Cognitive change was modelled as a continuous variable, and predictions are shown for values corresponding to the 25th ('less favorable': memory=-0.011 SD/year, fluency=0.000 SD/year), 50th ('median': memory=-0.004 SD/year, fluency=0.010 SD/year), and 75th ('more favorable': memory=0.004 SD/year, fluency=0.021 SD/year) percentiles of the cognitive change distribution. Estimates are derived from compositional models adjusted for age at wave 10 (2021-23), and the following baseline covariates: memory or fluency performance, sex, educational attainment, wealth, high blood pressure, diabetes, cancer, heart disease, stroke, psychiatric conditions, self-rated hearing, alcohol use, smoking status, self-reported physical activity, depressive symptoms, and number of mobility limitations.
Abbreviations: MVPA, moderate-to-vigorous physical activity; LPA, light physical activity; SB, sedentary behavior

© 2026 Bloomberg M et al. *JAMA Network Open*.

eReferences

1. Doherty A, Jackson D, Hammerla N, et al. Large Scale Population Assessment of Physical Activity Using Wrist Worn Accelerometers: The UK Biobank Study. *PLOS ONE* 2017; **12**(2): e0169649.
2. Chen Y, Chan S, Bennett D, et al. Device-measured movement behaviours in over 20,000 China Kadoorie Biobank participants. *International Journal of Behavioral Nutrition and Physical Activity* 2023; **20**(1): 138.
3. Walmsley R, Chan S, Smith-Byrne K, et al. Reallocation of time between device-measured movement behaviours and risk of incident cardiovascular disease. *British Journal of Sports Medicine* 2022; **56**(18): 1008-17.
4. Payton NM, Marseglia A, Grande G, et al. Trajectories of cognitive decline and dementia development: A 12-year longitudinal study. *Alzheimers Dement* 2022.
5. Sebaldt R, Dalziel W, Massoud F, et al. Detection of Cognitive Impairment and Dementia Using the Animal Fluency Test: The DECIDE Study. *Canadian Journal of Neurological Sciences* 2009; **36**(5): 599-604.
6. Turvey CL, Wallace RB, Herzog R. A Revised CES-D Measure of Depressive Symptoms and a DSM-Based Measure of Major Depressive Episodes in the Elderly. *International Psychogeriatrics* 1999; **11**(2): 139-48.
7. Vachon DD, Krueger RF, Irons DE, Iacono WG, McGue M. Are alcohol trajectories a useful way of identifying at-risk youth? A multiwave longitudinal-epidemiologic study. *Journal of the American Academy of Child & Adolescent Psychiatry* 2017; **56**(6): 498-505.
8. Berrington A, Smith P, Sturgis P. An overview of methods for the analysis of panel data. 2006.
9. Yang CH, Maher JP, Ponnada A, et al. An empirical example of analysis using a two-stage modeling approach: within-subject association of outdoor context and physical activity predicts future daily physical activity levels. *Transl Behav Med* 2021; **11**(4): 912-20.
10. Rajagopal SK, Beltz AM, Hampstead BM, Polk TA. Estimating individual trajectories of structural and cognitive decline in mild cognitive impairment for early prediction of progression to dementia of the Alzheimer's type. *Sci Rep* 2024; **14**(1): 12906.
11. Dumuid D, Stanford TE, Martin-Fernández J-A, et al. Compositional data analysis for physical activity, sedentary time and sleep research. *Statistical methods in medical research* 2018; **27**(12): 3726-38.
12. Van den Boogaart KG, Tolosana-Delgado R. “Compositions”: a unified R package to analyze compositional data. *Computers & Geosciences* 2008; **34**(4): 320-38.
13. Aitchison J. The statistical analysis of compositional data. *Journal of the Royal Statistical Society: Series B (Methodological)* 1982; **44**(2): 139-60.
14. Pasanen J, Leskinen T, Suorsa K, et al. Effects of physical activity intervention on 24-h movement behaviors: a compositional data analysis. *Scientific Reports* 2022; **12**(1): 8712.
15. Rasmussen CL, Palarea-Albaladejo J, Johansson MS, et al. Zero problems with compositional data of physical behaviors: a comparison of three zero replacement methods. *International Journal of Behavioral Nutrition and Physical Activity* 2020; **17**(1): 126.
16. Little RJ. Missing-data adjustments in large surveys. *Journal of Business & Economic Statistics* 1988; **6**(3): 287-96.

© 2026 Bloomberg M et al. *JAMA Network Open*.
