# Supplementary material for: Cognitive Trajectories and Subsequent Accelerometer-Measured Movement Behavior in Older Adults
Source: JAMA Netw Open. 2026 May 19;9(5):e2613399. doi: 10.1001/jamanetworkopen.2026.13399 (PMC13187880; doi:10.1001/jamanetworkopen.2026.13399)
Supplement: Supplement 2. — Data Sharing Statement [file jamanetwopen-e2613399-s002.pdf]

Data Sharing Statement

Bloomberg. Cognitive Trajectories and Subsequent Accelerometer-Measured Movement Behavior in Older Adults. *JAMA Netw Open*. Published May 19, 2026. doi:10.1001/jamanetworkopen.2026.13399

Data
Data available: Yes
Data types: Deidentified participant data
How to access data: ELSA data are available to researchers for non-commercial use upon registration with the UK Data Service at the following URL: https://datacatalogue.ukdataservice.ac.uk/series/series/200011#abstract
When available: With publication

Supporting Documents
Document types: None

Additional Information
Who can access the data: Any researcher requesting the data for non-commerical use
Types of analyses: Non-commercial use
Mechanisms of data availability: ELSA data are available to researchers for non-commercial use upon registering with the UK Data Service and signing the data access agreement.
